# Assessment of integrated community case management of childhood illness (ICCM) practices by trained patent and proprietary medicine vendors (PPMVs) in Ebonyi and Kaduna states, Nigeria

**DOI:** 10.1186/s12913-023-09067-6

**Published:** 2023-01-20

**Authors:** Akpu Blessing Oko, Anyanti Jennifer, Adizue Jane Chinyere, Nwankwo Jehosephat Nelson, Idogho Omokhudu, Onyezobi Chinedu Edward, Aizobu Dennis

**Affiliations:** Society for Family Health, Abuja, Nigeria

**Keywords:** Mortality, Under – 5, Caregiver, Community, Intervention, Malaria, Pneumonia and diarrhea

## Abstract

**Introduction:**

An Integrated Community treatment of Childhood disease (ICCM)- focused intervention involving a large number of Patent and proprietary medicine vendors (PPMVs) was conducted by Society for Family Health Nigeria to improve management of childhood, malaria, pneumonia and diarrhea with an intervention approach focused on knowledge and skill improvement. The intervention was conducted in Kaduna and Ebonyi state; recruited and trained 15 interpersonal communication agents (IPCAs) who were saddled with the responsibility to sensitize and mobilize caregivers with children within the age bracket of 2 months to 5 years to our mapped PPMVs within the communities, on the account of Malaria, Diarrhea, and Pneumonia; while the IPCAs in return monitor the quality-of-service delivery. Following the intervention, the Society for Family health conducted a study to demonstrate the effectiveness of interventions such as ICCM training, supervision and linkage to quality ICCM commodities, among PPMVs to achieve high levels of knowledge and performance in diagnosing and treating common childhood illnesses.

**Methods:**

Longitudinal research (before and after study) was adopted for the study. From the 387 PPMVs recruited and trained by SFH, 165 PPMVs were systematically selected to participate in the study, before and after the implementation of the intervention. Using SPSS version 22, data from the observation and completed questionnaires were analyzed and a chi-square test was used to examine the associations between the categorical information collected prior and after the intervention. The analysis was conducted at 5% level of significance.

**Results:**

More than 50 % of the study participants were females (56.4%) and majority were either Junior community extension workers (35%) or Senior community extension worker (27%). About 21.8% trained PPMVs could not appropriately treat malaria in the first quarter of the intervention, however, there was a significant decrease to 1.8% in second quarter in the number of those that cannot appropriately diagnose and treat malaria. There was also a decrease in the number of those who could not treat cough and fast breathing from 47(28.5%) to 14(8.5%) in the second quarter and for diarrhea from 33.3% in the first quarter to 2.4% in the second quarter.

**Conclusion:**

The study revealed a significant improvement in the quality of treatment provided by the trained PPMVs across the three disease areas. PPMVs in hard-to-reach areas should be trained and supported to continuously provide quality services to change the indices of under-5 mortality in Nigeria.

## Introduction

Child mortality records show that in 2020 1 in every 13 children die before age five in Sub Saharan Africa [2, 18]. In Nigeria, under five mortality rate is 132 per 1000 live births meaning that 1 in 8 Nigerian children never reaches the age of 5 [13]. Malaria, pneumonia and diarrhea are the leading cause of death among children under the age of 5 years especially in the rural and underserved setting in Nigeria. These diseases are responsible for approximately one third of global deaths among children under five in 2018 totaling an estimated 1.6 million deaths in this age group [11]. Nigeria accounts for almost 15% of global under-5 mortality [6].

In spite of being preventable and treatable with low-cost public health intervention, malaria, pneumonia and diarrhea still remain the major killer diseases for children under the age of 5 years with 23, 3, and 13% as prevalence respectively [13]. However, UNICEF reported pneumonia to be the highest killer of children under the age of 5 years in Nigeria, with 162,000 estimated deaths recorded in 2018.

The trio are diseases of inequity – one concentrated within the poorest populations around the globe with disproportionate prevalence among the poor and the rich. More so, every 2 minutes a child dies of Malaria, and in every 39 seconds a child dies of pneumonia [17]..

The most immediate identifiable cause of Nigeria’s high number of under 5 deaths is a service delivery gap that limits access to health services. Research has shown that infant mortality is a major problem in Nigeria and has been linked to factors such as inadequate health facilities, lack of financial capacity, and lack of access to appropriate medical care [3]. Problems such as cost of treatment, deplorable state of health facilities, distance to health facility, lack of awareness and knowledge for informed decisions and referral are some of the many difficulties stated by caregivers in describing difficulty with accessing healthcare [19]. These problems are particularly worrisome in the rural areas of developing countries where the density of modern health care facilities is low [1].

Nigeria’s progress in reducing child mortality has experienced a growing divide and inequality between rural and urban areas, as well as a concentration of under-five mortality in hard-to-reach communities [12]. One of the reasons given for this inequality in childhood mortality reduction is the growing inequality in access to health care across communities and regions [1]. There is also an uneven distribution of healthcare facility and shortage of human resource for health. More so, the poor health seeking behavior of caregivers of children under the age of 5 years also contributes significantly to the high infant and under-five mortality [10]. Although households and communities have a major responsibility in recognizing when children need treatment outside the home, a recent national survey found that this has not been the case due to poor health seeking knowledge and practices in households [4].

In response to the unacceptable high mortality rate of children under 5 years in Nigeria, the Federal Government developed several policies and programs to improve health and address the identified gaps. Some of them include National Child Health Policy, the National Malaria Policy, the Integrated Management of Childhood Illness Strategy and more recently the Integrated Maternal, Newborn and Child Health Strategy which is in line with the National Strategic Health Development Plan [14]. Integrated Management of Childhood Illness (IMCI) and ICCM are the key child survival thrust being used by the Nigerian Government to address the unacceptably high under-5 morbidity and mortality indices [19].

A recent systematic review of PPMV practices shows that PPMVs provide medicines and services for a wide variety of health needs, including malaria, respiratory infections, diarrhea [16]. The study also noted that an estimated 200,000 PPMVs operating in Nigeria are the first source of care for up to 55% of under-five child illnesses, such as malaria and diarrhea [16]. The evidence regarding the quality of services provided in the community by PPMVs who have been trained, mentored, and supervised to provide child health services is still limited despite the number of PPMVs, their presence in rural communities and markets, and their importance in improving primary health care in Nigeria.

Consequently, an ICCM- focused intervention which encompassed a large scale of PPMVs was conducted by a Nigeria based public health organization, Society for Family Health, to improve management of childhood, malaria, pneumonia and diarrhea with an intervention approach focused on knowledge and skill improvement through training and feedback and support through mentorship. This study seeks to demonstrate the effectiveness of interventions such as ICCM training, supervision and linkage to quality ICCM commodities, among PPMVs to achieve high levels of knowledge and performance in diagnosing and treating common childhood illnesses.

## Materials and methods

### Approach

*Delivering Health to all Children* (DELL2ALL) is a social and behavior change communication intervention funded by NORVATIS foundation and implemented by Society for Family Health (SFH). The intervention was designed to address equity gaps and expand access to life saving efforts which will lead to reduction in the unacceptable high mortality rate in children under the age of 5 years in the implementing states. The project was committed to building the capacity of Patent and Proprietary Medicine Vendors (PPMVs) in the implementing states. The PPMVs were saddled with the responsibility of providing timely treatment for common childhood illnesses (pneumonia, malaria and diarrhea) which cause the largest child mortality in Nigeria.

This intervention was implemented in Ebonyi and Kaduna State and designed in line with the Nigeria Federal Ministry of Health (FMoH) integrated community case management of childhood illness (ICCM) strategy. Ebonyi and Kaduna state were selected based on the high prevalence rate of malaria, pneumonia, diarrhea, and under 5 mortality rates both states. Ebonyi state is located in the Southeastern regions of Nigeria with over 3 million inhabitants spread across 13 administrative units referred to as Local Government Areas (LGAs) while Kaduna is a State in the Northwestern region of Nigeria serving as a home for more than 6 million residents with 23 LGAs. The intervention as well as the study was implemented in 4 LGAs across the two states; Ezza North and Ohaukwu LGAs in Ebonyi while Igabi and Sabongari LGAs in Kaduna.

The intervention commenced with the recruitment and training of 15 IPCAs in each state on Integrated Community Management of childhood illness (iCCM). These IPCAs are responsible for mobilizing to mobilize caregivers with children (2 – 59 months) to our mapped PPMVs on the account of malaria, diarrhea and penuemonia. They also monitor the quality of services delivery. A total 194 PPMVs were trained in Ebonyi state while 193 were trained in Kaduna State. These category of PPMVs mapped for this intervention were those with healthcare qualification (tier 2). The tier 1 PPMVs (those without healthcare qualification) and tier 3 (pharmacy technicians) were not included the programme.

### Study design

The study adopted a longitudinal research design (before and after study), targeted at the PPMVs under the intervention programme. A standard checklist and questionnaire were designed to observe providers (PPMVs) during the service provision. At the first quarter (first 3 months) of the intervention, the quality of services by the PPMVs were assessed using the standard checklist. The assessment was repeated towards the end of the second quarter (at the end of 6 months) and the two datasets were compared to determine the level of improvement in quality-of-service delivery.

### Sample size estimation

From the 387 PPMVs trained, the study sampled 165 PPMVs trained under Delivering Health to all Children program of the Society for family health. The sample size was determined using Yamane sample size formular

$$\boldsymbol{n}=\frac{\boldsymbol{N}}{\textbf{1}+\boldsymbol{N}\ast {\left(\boldsymbol{e}\right)}^{\textbf{2}}}$$

Where N is the total number of PPMVs trained under the Delivery Health to all Children programme (acceptable sampling error) = 0.05.

These 165 PPMVs were systematically selected from the pool of 387 PPMVs trained. The systematic sampling process is explained:

- We randomly assigned numbers from 1 to 387 to all the trained PPMVs.
- We divided the total number of trained PPMVs by the sample size (ie. 387/165 = 2.3 approximately 2.0)
- We randomly selected a number between 1 and 2 as the starting point and maintained a gap of 2 until all the 165 PPMVs were randomly selected.

### Study tool

Data was collected using structured questionnaires and observation checklists containing close ended questions which explored quality treatment practices and knowledge of integrated management of early childhood illnesses among the PPMVs. The observation tool was designed to observe if the caregivers were politely attend to and properly assessed for respiratory cough, diarrhea and fever. The observation tool also checked if the PPMV assessed some danger signs and make correct choice for drugs with the appropriate dosage. The questionnaire asked questions on data quality, availability of drugs, community involvement and water and sanitation.

### Data analysis

Data from the observation and completed questionnaires was analyzed using SPSS version 22. The results were presented using frequencies and proportions while associations between categorical variables were tested using Chi-square test (Test of Independence) at significance level of 0.05.

## Results

### Background characteristics

More than half of the 165 PPMVs sampled for the study were females (56.4%) as shown in Table 1. Kaduna state has the highest proportion of PPMVs assessed for the study (56%). All the PPMVs assessed had been exposed to some level of health training. More than a quarter of the PPMVs were trained nurses (26%), 27% were Senior Community Health Extension Workers (S. CHEW), 12% were midwifes and 35% were Junior Community Health Extension Workers (JCHEW).

**Table 1:** Background characteristics

Variable	Frequency (***n*** = 165)	Percent (%)
**LGA**		
Ezza North	47	29
Igabi	56	34
Ohaukwu	25	15
Sabon Gari	37	22
**Gender**		
Male	72	43.6
Female	93	56.4
**Qualification**		
Junior CHEW	57	35
Senior CHEW	45	27
Registered Nurse	43	26
Midwife	20	12

### Assessment of the quality of child health services offered among trained PPMVs

The capacity and willingness of the trained PPMVs to take respiratory rate were tested. In Table 2 below, the proportion of trained PPMVs who accurately took and frequently measure respiratory rate each time a kid was brought in the first quarter were 60.6%. After the intervention period, majority of the PPMVs (93.3%) were able to reliably measure the respiratory rate of a sick child (X^2^ (1) = 49.85, *p*-value < 0.001).

**Table 2:** Impact assessment of PPMVs training on quality of services offered to children

Variable	Observation	(***N*** = 165) Quarter 1 n (percent)	Quarter 2 n (percent)	df	𝜒^**2**^ (***p***-value)
**Took respiratory rate**	**Yes**	100 (60.6)	154 (93.3)	1	49.85 (0.000)
**Determined fast breathing**	**Yes**	89 (53.9)	151 (91.5)	1	58.73 (0.000)
**Observed chest in-drawing**	**Yes**	82 (49.7)	145 (87.9)	1	56.02 (0.000)

Also in the first quarter, 53.9% could accurately diagnose fast breathing in sick children while nearly all of the PPMVs could accurately diagnose fast breathing in sick children by the end of the intervention (X^2^ (1) = 58.73, *p*-value < 0.001). The number of PPMVs that check for chest in-drawing in unwell children before delivering treatment increased significantly from 49.7% in quarter 1 to 87.9% at the end of intervention (X^2^ (1) = 56.02, *p*-value < 0.001).

### Assessment of the quality of diagnosis for fever, cough, diarrhea

When the two quarters were compared in Table 3 to examine the quality of diagnosis for fever, cough, and diarrhea, it was discovered that there was an established gap in the number of PPMVs that assessed cough. In the first quarter, 18.2% of the PPMVs failed to ask for or assess cough in sick children, while only 7.3% failed to do so before treatment at the end of the intervention (𝜒^2^ = 8.84, *p*-value = 0.003). In the first quarter, 17.6% of those who assess or ask for cough did not take the respiratory rate; this number reduced to 12.7% in the second quarter (𝜒^2^ = 1.51, *p*-value = 0.219). In addition, only 30 (18.2%) of the PPMVs in this study were able to correctly count respiratory rate in the first quarter. In quarter 2, the result improved to 100(60.6%) (𝜒^2^ = 64.60, *p*-value < 0.001). Only 126 (76.4%) tested for diarrhea during the first phase of the assessment, but in the second phase, 154 (93.3%) adopted the correct practice of assessing under-5 children for diarrhea before treatment (𝜒^2^ = 18.48, *p*-value < 0.001). There was also a considerable improvement in the accuracy of fever diagnosis. In the first quarter of the trial, 24 (14.5%) of the PPMVs did not screen for fever before treating the child. According to the second quarter’s assessment, 159 (96.4%) of PPMVs examined if the child had a fever before treating them (𝜒^2^ = 11.88, *p*-value = 0.001). This finding also shows that in the first quarter, 40.6% of PPMVs did not perform RDT tests on children under the age of five who had a fever. In the second quarter, 87.3% of PPMVs were seen doing RDT tests before treatment (𝜒^2^ = 64.60, *p*-value = 0.000), indicating an improvement in diagnostic practice. At the start of the study, 71.5% of the PPMVs could not read the test result; by the second quarter, all (100%) of the PPMVs in the study could competently interpret the test result (𝜒^2^ = 183.68, *p*-value < 0.001). They were tested on their capacity to accurately identify all difficulties, and there was a substantial increase in the proportion of PPMVs who were unable to do so (𝜒^2^ = 0.34, *p*-value = 0.563).

**Table 3:** The quality of diagnosis for fever, cough, diarrhea

Variable	Observation	(***N*** = 165) Quarter 1 n (percent)	Quarter 2 n (percent)	𝜒^**2**^ (***p***-value)
**Does the CORP ask for or assess Cough?**	**Asked**	135 (81.8)	153 (92.7)	8.84 (0.003)
**Does the CORP assess or take respiratory rate? (for cough only)**	**Yes**	136 (82.4)	144 (87.3)	1.51 (0.219)
**Counted correctly**	**Yes**	30 (18.2)	100 (60.6)	64.60 (0.000)
**Does the CORP ask for or assess Diarrhea?**	**Asked**	126 (76.4)	154 (93.3)	18.48 (0.000)
**Does the CORP ask for or assess Fever?**	**Asked**	141 (85.5)	159 (96.4)	11.88 (0.001)
**Does the CORP assess or take RDT? (for fever only)**	**Yes**	98 (59.4)	144 (87.3)	32.79 (0.000)
**Interpreted correctly?**	**Yes**	47 (28.5)	165 (100)	183.68 (0.000)
**Does the CORP identify all problems correctly?**	**Yes**	134 (81.2)	138 (83.6)	0.34 (0.563)

### Assessment of the quality of treatment for fever, cough, diarrhea

During the intervention time, the PPMVs’ treatment quality in all three illness areas vastly improved according to the information on Table 4. In the first quarter, 21.8% of people did not know how to treat malaria; however, the percentage fell to 1.8% in the second quarter (𝜒^2^ = 31.67, *p*-value < 0.001). In the second quarter, the number of people who couldn’t treat cough and fast breathing dropped from 47 (28.5%) to 14 (8.5%) (𝜒^2^ = 21.90, *p*-value < 0.001). In the second quarter, the number of PPMVs that were unable to treat diarrhea after following the proper treatment protocol fell from 33.3 to 2.4% (𝜒^2^ = 53.68, *p*-value < 0.001). In the first quarter, 43.6% of PPMVs did not know what pre-referral treatment to administer, but this reduced to 14% in the second quarter (𝜒^2^ = 35.49, *p*-value < 0.001).

**Table 4:** The quality of treatment for fever, cough, and diarrhea

Variable	Observation	(***N*** = 165) Quarter 1 n (percent)	Quarter 2 n (percent)	𝜒^**2**^ (***p***-value)
**How to treat malaria?**	**Yes**	129 (78.2)	162 (98.2)	31.67 (0.000)
**How to treat cough and fast breathing?**	**Yes**	118 (71.5)	151 (91.5)	21.90 (0.000)
**How to treat diarrhea?**	**Yes**	110 (66.7)	161 (97.6)	53.68 (0.000)
**What pre-referral treatment to give?**	**Yes**	93 (56.4)	142 (86.0)	35.49 (0.000)

### Comparing trends in case management for common childhood illnesses among PPMVs

When case management for common childhood illnesses among PPMVs were examined between the two quarters, there was a significant shift as shown in Table 5. When a sick child is brought to their facility, the PPMVs have been habituated to looking for danger indications (𝜒^2^ = 2.59, *p*-value = 0.274). There was also an improvement in the practice of referring a child who showed signs of danger. In the second quarter, the number of PPMVs who would outright refer a child with a danger indication increased from 92(55.8%) to 156(94.5%) (𝜒^2^ = 66.47, *p*-value < 0.001). Improved referral facilitation and follow-up were also observed. In the second quarter, the percentage of improved referral facilitation and follow-up increased from 50.3 to 84.2% (𝜒^2^ = 43.16, *p*-value < 0.001). The response was considered “Yes” for all PPMVs that provided referral note, first dose of drugs, and counsel for follow-up correctly.

**Table 5:** Comparing case management for common childhood illnesses among PPMVs

Variable	Observation	(***N*** = 165) Quarter 1 n (percent)	Quarter 2 n (percent)	𝜒^**2**^ (***p***-value)
**Does the CORP ask or assess for danger signs?**	**Checked**	94 (57)	100 (60.6)	2.59 (0.274)
**Refers if child has a danger sign or condition he/she cannot treat?**	**Yes**	92 (55.8)	156 (94.5)	66.47 (0.000)
**Facilitate referral (provide referral note and first dose of drugs) and counsel for follow-up correctly**	**Yes**	83 (50.3)	139 (84.2)	43.16 (0.000)

### Assessment of improvement in data quality

There was an overall improvement in data gathering and record keeping. In Table 6, more PPMVs learned how to accurately fill out the daily CORP register. During the study period, the number of PPMVs who could fill daily corps registers increased from 144 (87.3%) to 156 (94.5%) (𝜒^2^ = 5.65, *p*-value = 0.059). Other than the 110(66.7%) documented in the first quarter, 132(80.0%) learned how to do their page summaries for the last full sheet (𝜒^2^ = 8.07, *p*-value = 0.018). They also made it a habit to preserve copies of the prior three ICCM HMIS forms. Only 125 (75.8%) possessed copies of their prior three ICCM HMIS forms in the first quarter, but 141 (85.5%) did in the second (𝜒^2^ = 29.39, *p*-value < 0.001). The importance of sending their ICCM HMIS forms to health facilities has also been recognized by PPMVs. Only 73 (44.2%) of PPMVs submitted an ICCM HMIS form to the health institution in the first quarter, but following months of skill reinforcement, 115 (69.7%) of PPMVs submitted an ICCM HMIS form to the health facility ((𝜒^2^ = 22.63, *p*-value < 0.001).

**Table 6:** Assessment of improvement in data quality

Variable	Yes	(***N*** = 165)	
	Quarter 1 n (percent)	Quarter 2 n (percent)	𝜒^**2**^ (***p***-value)
**Daily CORPs register filled correctly**	144 (87.3)	156 (94.5)	5.65 (0.059)
**Page Summaries done correctly for last full sheet**	110 (66.7)	132 (80.0)	8.07 (0.018)
**Copies of at least previous 3 ICCM HMIS forms kept**	125 (75.8)	141 (85.5)	29.39 (0.000)
**ICCM HMIS form submitted to health facility last month**	73 (44.2)	115 (69.7)	22.63 (0.000)

### Assessment of the availability of commodities

Table 7 shows that the intervention’s activities to create demand increased commodity demand. Because of this, the availability of several items has dropped, showing increased demand. Examples include amoxicillin (dropped from 77.6 - 63.0%), low osmolar ORS (from 92.1 - 91.5%), and antimalarials (from 89.1 - 81.2%) indicating increase in demand. The PPMVs’ connection to local medication manufacturers increased product availability. In the second quarter, the number of products available expanded dramatically. The number of PPMVs with at least 10 packs of 25 mg Arthemether – 67.5 mg Amodiaquine increased by 32.1% (𝜒^2^ = 44.85, *p*-value = 0.000), 50 mg Arthemether – 135 mg Amodiaquine increased from 41.8 to 66.1% (𝜒^2^ = 36.60, p-value < 0.001), while the number of PPMVs with roughly 60 Zinc pills increased from 88.5 to 90% (𝜒^2^ = 0.29, *p*-value = 0.592). The number of PPMVs with a timer and a continuous supply of AL/AA, Amoxicillin, and Low Osmolar ORS for 7 days or more without any stock-out of this product increased from 8.5 to 90.9% (𝜒^2^ = 1.81, p-value = 0.404).

**Table 7:** Availability of commodities

Variable	Yes	(***N*** = 165)	
	Quarter 1 n (percent)	Quarter 2 n (percent)	𝜒^**2**^ (***p***-value)
**Dispersible amoxicillin available till the last day of the month.**	128 (77.6)	104 (63.0)	8.48 (0.014)
**Arthemether Lumefantrine (AL) at least 20 full dose**	134 (81.2)	135 (81.8)	0.34 (0.845)
**25 mg Arthemether – 67.5 mg Amodiaquine (at least 10 packs)?**	63 (38.2)	116 (70.3)	44.85 (0.000)
**50 mg Arthemether- 135 mg Amodiaquine (at least 10 packs)?**	69 (41.8)	109 (66.1)	36.60 (0.000)
**Low osmolar ORS (at least 10 sachets)**	152 (92.1)	151 (91.5)	0.04 (0.841)
**Antimalarial drugs available till the last day of the month.**	147 (89.1)	134 (81.2)	4.05 (0.044)
**Low osmolar ORS available till last day of the Month.**	148 (89.7)	143 (86.7)	0.73 (0.394)
**Zinc tablet (approximately 60 tablets)**	146 (88.5)	149 (90.3)	0.29 (0.592)
**Chlorhexidene gel 4% (at least 10 tubes)**	14 (8.5)	23 (13.9)	62.64 (0.000)
**Continuous supply of AI/AA, Amoxicillin and Low Osmolar ORS for the last 3 months without any stock out of this product.**	106 (64.3)	58 (35.2)	1.18 (0.555)
**Respiratory timer available, and a continuous supply of AI/AA, Amoxicillin and Low Osmolar ORS For 7 Days or more without any stock-out of this product**	14 (8.5)	150 (90.9)	1.81 (0.404)

## Discussion

This research focuses on reducing under-5 mortality rate resulting from malaria, pneumonia, and diarrhea in Nigeria by improving the skill of PPMVs to offer high-quality, cost-effective health care to under-5 children with malaria, pneumonia, or diarrhea. At the outset, the quality of the services was subpar, however the training and implementation of the DEL2ALL interventions increased service quality. This is in line with prior research, which showed that increasing ICCM for uncomplicated pneumonia with the help of PPMVs can help to lower disease burden [14].

Late detection of childhood illnesses is a leading cause of death in underdeveloped nations, according to recent studies. Early detection and treatment of illnesses will reduce the severity of cases and fatality.. This study shows that if PPMVs are given diagnostic supplies and pediatric formulations, instances will be identified earlier and diseases in children will be better handled. In a recent study in Uganda, drug retailers who were taught in ICCM and given diagnostic supplies as well as pre-packaged kid versions of ACT, amoxicillin, ORS, and zinc successfully managed childhood infections in 87.7% of cases [5].

To engage PPMVs in delivering ICCM, more training is required to improve fundamental health awareness and stocking and selling standards. The WHO recommends continuing education and promotion of ICCM through PPMVs in an evidence-based guideline for the management of diseases in children under the age of five [19]. Through training and support of PPMVs, promotion of ICCM for the treatment of uncomplicated cases of malaria, pneumonia, and diarrhea in children under the age of 5 years offers promise.

PPMVs are qualified to train and certify as community-based resource persons to deliver ICCM. The training provided before enlisting PPMVs to deliver ICCM, as well as the ongoing assistance improved PPMVs’ case management capacity for common childhood illnesses. In some places, ICCM activities have been successful owing to the participation of trained community health workers. In Ghana and Uganda, lay community health workers who had been trained in dosage and referral for fever and pneumonia treatment were able to treat the majority of cases among children under the age of five [8].

PPMVs also have a number of attributes that appeal to community members, including longer opening hours, friendliness, and more consistent availability than public facilities, which are prone to service outages [7, 15]. Because of their broad presence, well-regulated and well-trained PPMVs could help Nigeria achieve Universal Health Coverage by becoming increasingly involved in innovative health initiatives like integrated community case management (ICCM). There was a general improvement in record keeping and data collecting as a result of this research. More PPMVs learned how to accurately fill out the daily corps register. If properly trained, PPMVs could function as an efficient lay cadre of ICCM providers in Nigeria. During the intervention period of this study, the quality of therapy provided by the PPMVs in all three disease areas significantly improved, due to the trainings and support.

Our findings show that PPMV trainings are still an effective way to help PPMVs enhance their capacity to provide better healthcare to their communities. Increasing the frequency of training can help improve product distribution accessibility and coverage [9]. The assistance offered through the DEL2ALL method enhanced commodity access and availability. Well trained PPMVs are an excellent platform for optimum healthcare delivery since they stock and sell products and have appropriate understanding of the necessary therapy, and this is dependent on their ongoing training.

Refresher trainings for PPMVs have the impact of enhancing the level of knowledge and practices in specific areas, such as acceptable prescription procedures for the treatment of diarrhea, malaria, and pneumonia. Drug stores play an essential role in delivering basic healthcare services in Nigeria, and interventions to reduce disease and death must take this into account.

## Conclusion

According to the study, the trained PPMVs were significantly better at treating the three types of diseases. It demonstrates how, if given the right instruction and assistance, PPMVs can serve as a significant source of health care for children in areas with poor access to medical facilities. This cadre of healthcare professionals would be helpful in enhancing national statistics on under-5 mortality because to the substantial presence of PPMVs in rural and suburban populations. The provision of services will be considerably enhanced, and mortality will be reduced, by training PPMVs and enhancing their skills. By helping PPMVs connect with local drug producers and creating a system for them to stock commodities through the government, the issue of commodity supply and access can be resolved.

The PPMVs need undergo continuous training in order to help them understand their limitations and advance their knowledge of and use of early referral. Building PPMVs’ capacity should concentrate on improving their capabilities in the areas of pharmaceutical product knowledge and minor disease diagnostics. To discover the most effective training methods for enhancing PPMV knowledge, more research is required.

## Data Availability

The data that support the findings of this study are available from Society for family Health Nigeria but restrictions apply to the availability of these data, which were used under license for the current study, and so are not publicly available. Data are however available from the corresponding author upon reasonable request and with permission of the Society for Family Health Nigeria.

## Abbreviations

AA: Arthemether**-** Amodiaquine
ACT: Artemisinin- base Combination Therapy
AL: Arthemether Lumefantrine
CORPs: Community Resource Persons
DEL2ALL: Delivering Health to All Children
FMOH: Federal Ministry of Health
HMIS: Health Management Information System
ICCM: Integrated Community Case Management
IMCI: Integrated Management of Childhood Illnesses
IPCA: Interpersonal Communication Agents
JCHEW: Junior Community Health Extension Workers
LGA: Local Government Area
NDHS: National Demographic Health Survey
ORS: Oral Rehydration Solution
PPMV: Patent and Proprietary Medicine Vendors
RDT: Rapid Diagnostic Test
SCHEW: Senior Community Health Extension Worker
SFH: Society for Family Health
SPSS: Statistical Package for Social Sciences
UNICEF: United Nations International Children’'s Emergency Fund
WHO: World Health Organization
